# The dual role of autophagy in cartilage degradation: from mechanisms to targeted therapeutics

**DOI:** 10.3389/fcell.2026.1737547

**Published:** 2026-02-04

**Authors:** Jiahua Mei, Shenghao Zhang, Xinrong Cui, Ruiping Yang, Jin Ke, Lili Cui, Lin Tan, Shan Zhu, Yunshu Ma

**Affiliations:** 1 Yunnan University of Chinese Medicine, Kunming, China; 2 The Key Laboratory of External Drug Delivery System and Preparation Technology in University of Yunnan Province, Kunming, China; 3 Yunnan Key Laboratory of Dai and Yi medicines, Kunming, China

**Keywords:** autophagy, cartilage degeneration, cellular apoptosis, chondrocytes, osteoarthritis

## Abstract

Autophagy is a highly conserved cellular degradation and recycling process that plays a pivotal role in maintaining cartilage homeostasis. Normal autophagy is essential for the survival of chondrocytes and the preservation of the extracellular matrix (ECM); however, a decline in autophagic function may lead to the accumulation of damaged organelles and macromolecules, thereby reducing chondrocyte vitality and promoting apoptosis, which in turn contributes to the development of osteoarthritis (OA). This review summarizes the biological processes of autophagy, the interaction between autophagy and cartilage degeneration, as well as the interplay between autophagy and cellular senescence, apoptosis, inflammation, and oxidative stress. Furthermore, we explore key autophagic targets for the regulation of OA and discuss autophagy-targeting therapies, including mTOR inhibitors, AMPK activators, and natural products that target autophagy, along with emerging strategies aimed at modulating autophagy. Finally, the article highlights the challenges in the development of autophagy-targeting drugs for OA treatment and presents important scientific issues that warrant further investigation to guide future research.

## 1 Introduction

Osteoarthritis (OA) is a prevalent chronic, degenerative joint disorder characterized by the deterioration of articular cartilage, subchondral bone sclerosis, and the formation of osteophytes (Glyn-Jones et al., 2015). Upon onset, individuals experience pain, stiffness, and restricted joint function, profoundly impairing their quality of life (Martel-Pelletier et al., 2016). The global incidence of OA continues to rise, with a notably higher prevalence among middle-aged and elderly individuals, increasing significantly with age (Dell’Isola et al., 2025). Its pathogenic factors are complex, resulting from the interplay of mechanical, biological, and inflammatory influences (Barnett, 2018). Treatment options for OA include intra-articular injections and pharmacological therapies, yet their efficacy remains limited. Therefore, it is of paramount importance to further investigate the pathogenesis of OA, explore early preventive strategies, and develop novel, safe, and effective therapeutic approaches.

Cartilage degeneration is considered the primary pathological change at the tissue level associated with OA symptoms. Articular cartilage is primarily composed of chondrocytes and the extracellular matrix (ECM), the latter of which includes type II collagen and proteoglycans (Becerra et al., 2010). Chondrocytes are the sole cell type within cartilage, responsible for synthesizing and maintaining the balance of matrix components. They ensure the integrity and function of cartilage through a dynamic equilibrium between anabolic and catabolic processes (Komori, 2020). When the function of chondrocytes is impaired, a decline in anabolic activity coupled with an increase in catabolic processes leads to the degradation of the matrix. This breakdown of the matrix further alters the state of chondrocytes, ultimately resulting in their apoptosis and the complete disruption of articular cartilage homeostasis (Zhang, 2015). Therefore, articular chondrocytes play a crucial role in maintaining cartilage homeostasis.

Studies have revealed that the degeneration of articular chondrocytes may be linked to autophagy, which directly or indirectly influences the progression of OA (Chang et al., 2013). Autophagy is a highly conserved self-degradation and recycling process that occurs under cellular stress, removing damaged organelles and misfolded proteins to provide energy and raw materials, thereby maintaining cellular homeostasis (Liu et al., 2023). In OA, chondrocyte autophagy plays a dual role. On one hand, moderate autophagy clears dysfunctional mitochondria to alleviate oxidative stress and degrades abnormal protein aggregates to reduce endoplasmic reticulum stress (ERS), thus protecting the cartilage matrix. On the other hand, when various factors disrupt autophagic function, it promotes chondrocyte apoptosis and matrix degradation, thereby accelerating OA progression (McHugh, 2022). Mechanistically, dysfunctional autophagy can lead to detrimental outcomes through multiple pathways: (1) Impaired clearance of damaged mitochondria results in excessive reactive oxygen species (ROS) production and cytochrome C release, activating the caspase cascade and apoptosis (Pickrell and Youle, 2015). (2) Failure to degrade misfolded proteins aggregates exacerbates ERS, triggering the unfolded protein response and apoptotic pathways (Hetz, 2012). (3) Accumulation of dysfunctional autophagosomes or impaired lysosomal degradation can lead to the release of catabolic enzymes and calcium crystals, directly degrading the ECM and promoting pathological calcification (Varga et al., 2016). Therefore, regulating the autophagic activity of chondrocytes to restore their normal protective function is of vital importance for maintaining cartilage homeostasis and potentially delaying or halting the progression of OA.

This review is based on a systematic literature search conducted across PubMed, Web of Science, and Google Scholar databases using key terms including “autophagy” “chondrocytes” “osteoarthritis” “cartilage degradation” “mTOR” “AMPK” “ncRNA” and “senescence” Articles published between 2000 and 2025 were screened for relevance, with inclusion based on their direct contribution to understanding autophagy mechanisms or therapeutic strategies in OA. Building on this methodologically rigorous approach, we provide an integrated analysis that systematically summarizes the biological processes of autophagy and their multifaceted interactions with cartilage degeneration, apoptosis, inflammation, and oxidative stress in OA. We further evaluate key autophagic regulators as potential therapeutic targets and discuss emerging autophagy-modulating strategies. In addition, we critically examine the translational challenges and unresolved scientific issues in developing autophagy-targeted therapies for OA, with the aim of guiding future research directions.

## 2 The biological process of autophagy

Autophagy is a process through which cells clear damaged organelles, misfolded proteins, and other cellular debris, breaking them down into amino acids and other components, which are then used to synthesize new cellular constituents, thereby supporting normal cellular physiological functions (Choi et al., 2013). In mammalian cells, autophagy is classified into three types: macroautophagy, microautophagy, and chaperone-mediated autophagy (CMA). The common feature among these processes is the transport of materials to the lysosome for degradation and recycling. Macroautophagy, the most prevalent form, is referred to simply as autophagy in this review. It involves the formation of autolysosomes with a double-membrane structure to degrade a large volume of cellular contents. Microautophagy, in contrast, directly engulfs smaller amounts of cellular material through the invagination of the lysosomal membrane for degradation. CMA is highly specific to certain soluble proteins. It is characterized by the recognition and binding of target proteins by molecular chaperones, which then transport the proteins to receptors on the lysosomal membrane. Once bound, the target proteins are translocated into the lysosome for degradation, demonstrating a high degree of substrate specificity (Li et al., 2024).

**FIGURE 1:**
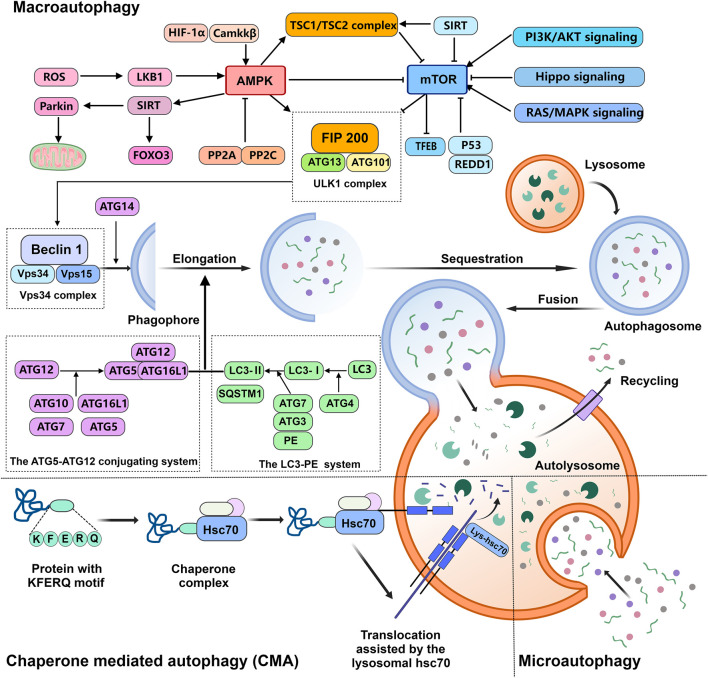
Mechanism of autophagy in OA chondrocytes. Autophagy in OA chondrocytes involves several types of autophagic processes, including macroautophagy, microautophagy, and chaperone-mediated autophagy. This article primarily focuses on macroautophagy, which is the most extensively studied form. Autophagy is a cellular process through which the cell degrades its own structures or materials to recycle nutrients in the cytoplasm, fulfilling metabolic requirements and renewing specific organelles. Autophagy is a multi-step process that relies on a series of specific protein complexes to complete, including the ULK1 complex and Vps34 complex. The transition from the phagophore to the autophagosome is mediated by two ubiquitin-like conjugation systems: the ATG5-ATG12 conjugation system and the LC3-PE system. In mammalian cells, mTOR and AMPK are the key regulators of autophagy in OA chondrocytes. Among these, mTOR acts as a negative regulator of autophagy, while AMPK serves as a positive regulator, promoting the activation of the autophagic process under stress conditions.

The process of autophagy involves a series of key steps: autophagy initiation, formation and elongation of the phagophore, autophagosome formation, autolysosome formation, and the degradation and recycling of materials (Mizushima et al., 2008). Among these, the formation of the autophagosome is the central step in the entire autophagic process. Autophagosome formation consists of initiation, nucleation, extension, and closure, and it relies on the coordinated action of four major protein complexes. The first step is initiation, which involves the ULK1 complex, composed of ULK1 kinase, autophagy-related genes (ATG) 13, RB1CC1/FIP200, and C12orf44/ATG101. Under nutrient-rich conditions, the mTORC1 complex interacts with the ULK1 complex, phosphorylating and inactivating ULK1 and ATG13. When stress signals arise, mTORC1 activity on the lysosomal membrane is suppressed, leading to decreased phosphorylation of ULK1 and ATG13, thus activating the ULK1 complex and initiating autophagy (Kim and Lee, 2014). The second step is nucleation, which involves the Vps34 complex, consisting of Beclin1, Vps34, Vps15, and ATG14. This complex mediates the formation of the pre-autophagosomal structure and recruits the ATG5-ATG12 conjugation system and LC3-PE system to facilitate phagophore extension. Additionally, the Vps34 complex is positively regulated by UVRAG, Grb2-like adapter protein B1, and Beclin1-regulating factor 1. Conversely, binding of Bcl2 to Beclin1 inhibits the formation of the complex and thus suppresses autophagy (Kroemer, 2015). The third step involves the extension of the phagophore, which is dependent on two ubiquitin-like modification processes: the ATG5-ATG12 conjugation system and the LC3-PE system. First, ATG12 is activated by ATG7, transferred and conjugated to ATG5 by ATG10, and then forms a complex with ATG16L1. This complex exhibits E3 ubiquitin ligase-like activity and promotes the transfer of ATG8/LC3 to membrane-associated phosphatidylethanolamine (PE), contributing to the expansion of the pre-autophagosomal membrane (Melino et al., 2019). Concurrently, the microtubule-associated protein light chain 3 (LC3) precursor is processed by ATG4 into soluble LC3-I, and, under the action of ATG7 and ATG3, is converted into lipid-soluble LC3-II, which participates in membrane extension (Antonioli et al., 2017). Finally, the autophagosome, assisted by the microtubule cytoskeleton and the SNARE protein superfamily as well as monomeric GTPase families, fuses with the lysosome to form the autolysosome. Proteins involved in this process include LAMP1, LAMP2, and UVRAG. Subsequently, the materials within the autophagosome are transported to the lysosome for degradation and recycling. The mechanism of autophagy in OA chondrocytes is illustrated in Figure 1.

## 3 Interaction between autophagy and cartilage degeneration

In avascular cartilage tissue with a low cellular metabolic rate, autophagy is crucial for the survival and functional maintenance of chondrocytes, serving as a core regulatory mechanism for cartilage homeostasis (Kao et al., 2022). In the early stages of OA, autophagy is activated at the surface of cartilage, likely as a response to cellular stress, in order to prevent cell death (Almonte-Becerril et al., 2010). However, in the late stages of OA, the expression of autophagy markers in OA cartilage and chondrocytes significantly decreases, including ULK1, Beclin1, LC3, ATG3, ATG5, and ATG12. This reduction in autophagic activity accelerates cartilage degeneration.

### 3.1 The role of autophagy in cartilage homeostasis

When chondrocytes are exposed to a hypoxic environment, their mitochondria are particularly vulnerable to dysfunction due to factors such as inflammation and mechanical stress, leading to the production of large amounts of ROS. This results in oxidative stress, exacerbating chondrocyte damage and extracellular matrix (ECM) degradation. Autophagy plays a critical role in mitigating this damage by specifically identifying and clearing dysfunctional mitochondria, thus reducing ROS production. For example, the PINK1/Parkin pathway is a classic mitophagy pathway. When the mitochondrial membrane potential is compromised, PINK1 recruits Parkin, which subsequently targets the damaged mitochondria for engulfment by the autophagosome and their removal, thereby protecting chondrocytes from damage (Li Q. et al., 2025). Moreover, during the progression of OA, inflammatory cytokines disrupt the chondrocytes’ ability to synthesize ECM, causing an accumulation of misfolded proteins in the endoplasmic reticulum (ER). This results in ERS, which can trigger apoptosis. Autophagy is activated in response to ER stress and helps clear these protein aggregates, alleviating the burden on the ER, restoring cellular function, and preventing chondrocyte apoptosis, thereby preserving the cells’ synthetic capabilities (Lu et al., 2024).

### 3.2 The role of autophagy dysfunction in cartilage degeneration

#### 3.2.1 Lipotoxic mechanism in cartilage degeneration

In OA, factors such as synovial inflammation contribute to elevated levels of free fatty acids (FFAs) in the joint. Under normal conditions, autophagy can degrade these excess lipids, maintaining cellular homeostasis. However, when autophagic function is impaired, FFAs accumulate within chondrocytes, where they are oxidized by mitochondria into toxic lipid metabolites like ceramides. These metabolites trigger mitochondrial dysfunction, causing increased ROS production, which further induces cytochrome C release and activates the caspase pathway, ultimately leading to chondrocyte apoptosis (Zhang, 2015). Studies have shown that in mice fed a high-fat diet or chondrocytes exposed to FFAs, autophagic activity is suppressed, resulting in the accumulation of interferon-induced gene stimulator 1 (STING1). This activation leads to the stimulation of the STING1-TBK1-IRF3 and MAP2K3/MKK3-MAPK/p38 signaling pathways, both of which trigger ECM degradation in cartilage (Kang et al., 2025). Furthermore, cholesterol homeostasis plays a crucial role in skeletal development, and dysregulated cholesterol levels have been linked to the progression of cartilage diseases, including OA. When autophagic function is impaired, the ability to clear intracellular cholesterol is reduced. Excess cholesterol becomes oversaturated, forming tiny cholesterol crystals that can be recognized by the NOD-like receptor pyrin domain-containing 3 (NLRP3). The activation of NLRP3 promotes the release of pro-inflammatory cytokines, creating a local inflammatory environment that accelerates ECM degradation and chondrocyte death (Papathanasiou et al., 2021). Research has demonstrated that five cholesterol-lowering drugs can enhance cholesterol efflux, effectively preventing cartilage degeneration. This suggests that restoring proper lipid and cholesterol homeostasis through targeted therapies could offer a promising approach to mitigating the progression of OA (Lee et al., 2025).

#### 3.2.2 Autophagy-calcium-calcification-degeneration vicious cycle in OA

Pathological cartilage calcification plays a crucial role in the progression of OA. The final step of the autophagic process involves the fusion of autophagosomes with lysosomes to form autolysosomes, where degradation occurs. However, when fusion fails or lysosomal function is impaired, undegraded autophagosomes accumulate within the cell. Research has shown that these stalled autophagosomes are rich in calcium, phosphate ions, and osteogenic differentiation markers (Pei et al., 2022). Upon cell death, these contents are released into the extracellular matrix (ECM), becoming the core of pathological calcification and promoting the formation of hydroxyapatite crystals, which further contribute to the mechanical wear of cartilage. In the early stages of OA, microtubule destabilization by histone deacetylase six hinders the fusion of autophagosomes with lysosomes, causing calcium-containing vesicles labeled by LC3 to be released into the matrix. This process triggers pathological calcification and accelerates cartilage degradation (Yan et al., 2022). Moreover, normal autophagy inhibits the abnormal differentiation of chondrocytes by degrading runt-related transcription factor 2 (Runx2). When autophagy is suppressed, proteins like Runx2 accumulate in the cell, driving the pathological phenotypic transformation of chondrocytes. These transformed cells exhibit osteoblast-like characteristics, such as high expression of alkaline phosphatase (ALP) and secretion of type I collagen rather than type II collagen, actively promoting subchondral bone sclerosis and cartilage matrix calcification. This disruption undermines the mechanical and lubricating properties unique to cartilage (Komori, 2022).

### 3.3 Environmental factors leading to autophagy imbalance

#### 3.3.1 Age-related decline in autophagy

Aging significantly impacts autophagy-mediated chondrocyte vitality. Studies have shown that autophagy-related proteins are highly expressed in normal chondrocytes, whereas their expression is substantially reduced in elderly populations, leading to a marked decrease in autophagic activity (Caramés et al., 2010). Concurrently, with advancing age, the number of lysosomes and their enzymatic activity decline, severely impairing the fusion efficiency of autophagosomes with lysosomes, as well as the degradation of autophagic substrates. Even when autophagosomes form properly, effective recycling cannot be achieved (Fujimaki et al., 2019). The diminished autophagic activity results in the accumulation of damaged organelles and macromolecules within the cells, ultimately compromising chondrocyte vitality and triggering age-related OA (Bouderlique et al., 2016).

#### 3.3.2 The dual effects of mechanical overload

Under mechanical stimulation, moderate stress can upregulate the expression of autophagy-related proteins, inducing protective autophagy and aiding in the clearance of damaged molecules produced during the mechanical stress (Vinatier et al., 2018). However, excessively high-intensity stimuli suppress autophagic activity, leading to cellular apoptosis (Cecconi and Levine, 2008). Additionally, when prolonged stress exceeds the cell’s compensatory capacity, mitochondrial damage occurs in chondrocytes, resulting in a significant increase in ROS and ultimately triggering OA (López de Figueroa et al., 2015). Furthermore, studies have reported that during physiological activities, the expression of the cartilage-specific transcription factor SOX9 in circulating mesenchymal progenitor cells, associated with autophagy, is elevated, suggesting the beneficial role of physical exercise in protecting cartilage formation (Dalle Carbonare et al., 2019).

#### 3.3.3 The role of circadian rhythm in cellular autophagy

Clinical and epidemiological evidence increasingly associates circadian rhythm disturbances with heightened OA risk and severity, positioning circadian disruption as a modifiable environmental and genetic risk factor. Shift work chronically disrupts endogenous rhythms and correlates with a higher prevalence of knee OA and accelerated joint degeneration (Berenbaum and Meng, 2016). Sleep disorders such as insomnia and obstructive sleep apnea, often accompanied by circadian misalignment, are common in OA patients and may exacerbate pain and functional impairment (Sánchez Romero et al., 2022). Additionally, genetic polymorphisms in core clock genes (*BMAL1*, *CLOCK*) have been linked to OA susceptibility and progression in human cohorts, underscoring the translational relevance of circadian biology in OA (Yu et al., 2019). Mechanistically, disruption of circadian rhythms impairs the temporal orchestration of autophagy, leading to dysfunctional autophagic flux in chondrocytes. Core clock genes, including BMAL1 and CLOCK, directly regulate the transcription of several autophagy-related genes and key autophagy regulators such as TFEB and FOXO3, whose expression exhibits circadian oscillations (Dudek et al., 2016). BMAL1, in particular, binds to enhancer box sequences in the promoters of various ATG genes, driving their rhythmic expression (Dudek et al., 2016). In cartilage-specific BMAL1 knockout mice, loss of rhythmic transcriptional activation of downstream autophagy genes reduces autophagic activity, leading to accelerated cartilage degeneration (Dudek et al., 2016). Circadian misalignment dampens these transcriptional rhythms, reduces basal autophagic activity, and impairs the clearance of damaged organelles and protein aggregates. Moreover, circadian disruption alters the circadian gating of mTOR and AMPK signaling pathways, further compromising autophagy induction and progression (Zhang W. et al., 2022). Other core circadian components also modulate autophagy: the CLOCK: BMAL1 heterodimer drives the expression of period (PER) and cryptochrome (*CRY*) genes, which feedback to inhibit CLOCK: BMAL1 activity. PER/CRY complexes interact with autophagy regulators; for instance, CRY1 stabilizes the autophagy-initiating ULK1 complex, and its loss leads to premature autophagy activation (Lane et al., 2023). Conversely, REV-ERBα/β repress the transcription of several autophagy and lysosomal genes by binding to RORE elements in their promoters, and pharmacological activation of REV-ERBα suppresses excessive autophagy and ameliorates tissue damage in inflammatory conditions (Lee and Kim, 2013). In chondrocytes, coordinated expression of these circadian genes ensures temporally controlled autophagy, optimizing cellular cleanup and energy homeostasis. Disruption of any component can lead to autophagic imbalance, contributing to OA pathogenesis through the accumulation of cellular debris, enhanced oxidative stress, and accelerated chondrocyte senescence and apoptosis, thereby driving cartilage degradation (Dudek et al., 2017).

## 4 The interrelationship between cellular autophagy and other pathological processes in OA

### 4.1 Cellular autophagy and senescence

Cellular aging refers to the process in which a cell’s proliferative and differentiative abilities, along with its physiological functions, gradually decline (Loeser et al., 2016). Key characteristics of cellular aging include the secretion of senescence-associated secretory phenotype (SASP) factors, loss of proliferative capacity, and DNA damage (Teh et al., 2024). The release of SASP factors has been shown to exacerbate mitochondrial dysfunction and trigger cellular oxidative stress, thereby inducing cellular senescence (Kuźniar et al., 2025). In functional cells, autophagy can suppress the activity of SASP by clearing its key regulatory factor, GATA binding protein 4, thereby delaying or preventing cellular aging. For example, Hu (Hu et al., 2022) found that cyclosporine A could inhibit the release of SASP induced by Hypoxia/Reoxygenation, suppress cellular senescence, and upregulate autophagy protein levels. Thus, there exists a negative regulatory relationship between autophagy and cellular senescence. During the pathogenesis of OA, a large number of senescent chondrocytes and synovial cells are produced, and the autophagic process in senescent chondrocytes gradually decreases, triggering cellular aging and accelerating the progression of OA (Chen X. et al., 2022). Liao (Lu et al., 2024) and colleagues discovered that Sr ions, through the AMPK/mTOR/LC3B-II signaling axis, improve the autophagic function of fibroblast-like synovial cells and delay their senescence, thereby alleviating OA.

### 4.2 Cellular autophagy and apoptosis

Apoptosis is a specific form of programmed cell death that interacts with autophagy. Chondrocyte apoptosis is a crucial factor in the progression of OA. Under normal conditions, autophagy in chondrocytes can suppress apoptosis and degradation, thereby inhibiting cartilage degeneration (Zhang et al., 2025c). However, when autophagy is chronically overactivated, it can selectively degrade mitochondria or cause extensive self-digestion, leading to autophagic cell death or promoting apoptosis, thus accelerating cartilage degeneration (Ristic et al., 2021). This dual effect indicates that, in the early stages of cartilage degeneration, moderate autophagy clears damaged mitochondria and misfolded proteins, suppresses apoptotic signals, and promotes chondrocyte survival under stress. For instance, mesenchymal stem cell-exosomes mediated long non-coding RNA (lncRNA) KLF3-AS1 can inhibit IL-1β-induced chondrocyte autophagy by activating the PI3K/Akt/mTOR signaling pathway, thereby promoting apoptosis. However, as cartilage degeneration intensifies and autophagy becomes severely dysregulated, excessive or defective autophagy can itself transform into pro-apoptotic signals (Wen et al., 2022). For example, the accumulation of undegraded autophagic vacuoles can activate caspase-8, or dysfunctional mitochondria can release large amounts of cytochrome C, ultimately synergizing to promote chondrocyte apoptosis, accelerating the loss of chondrocyte numbers, and exacerbating OA progression (Caramés et al., 2015).

### 4.3 Cellular autophagy and inflammatory response

Autophagy exerts anti-inflammatory effects by clearing the cytoplasm and activating the cell’s intrinsic inflammatory response. At the same time, inflammatory responses can suppress chondrocyte autophagy, reducing the autophagic rate (Zheng et al., 2021). In OA, inhibiting the PI3K/AKT/mTOR signaling pathway can promote autophagy in rat articular chondrocytes, alleviating inflammation (Xue et al., 2017). Additionally, sprouty-related EVH1 domain-containing protein two regulates the p38 MAPK signaling pathway, promoting chondrocyte autophagy in rats while inhibiting inflammation (Wei et al., 2023). Thus, a bidirectional regulatory relationship exists between autophagy and inflammation. Properly functioning autophagy controls inflammation by degrading NLRP3 and its upstream signaling molecules, preventing the excessive production of potent pro-inflammatory cytokines like IL-1β. Conversely, in the presence of autophagic defects, inflammatory signals cannot be cleared in a timely manner, leading to sustained activation of NLRP3 and exacerbating joint inflammation. The released inflammatory factors then suppress protective autophagy, creating a vicious cycle of inflammation and autophagic dysregulation, which jointly drives cartilage degeneration (Sasaki et al., 2012).

### 4.4 Cellular autophagy and oxidative stress

During cellular metabolism, mitochondria generate ROS and hydroxyl radicals, leading to oxidative stress (Kuźniar et al., 2025). ROS play a role in inducing autophagy by regulating the AMPK/mTORC1 and MAPK signaling pathways (Ristic et al., 2021). A specialized form of autophagy, mitophagy, eliminates damaged mitochondria, thereby reducing ROS production at its source and alleviating oxidative stress. In OA, factors such as aging and inflammation disrupt mitochondrial function, leading to the release of excessive ROS. For instance, IL-1β stimulates chondrocytes to produce ROS in excess, resulting in the accumulation of damaged mitochondria and mitochondrial dysfunction (Wang et al., 2023). Under normal autophagic conditions, excess ROS can be efficiently cleared, maintaining oxidative balance. However, when cells are damaged and autophagic function declines, ROS cannot be cleared promptly, causing a dramatic increase in oxidative stress. High levels of ROS not only directly damage proteins, lipids, and DNA but also further suppress autophagy, promoting inflammation and apoptosis, thereby establishing a vicious cycle of autophagy and oxidative stress in OA (López de Figueroa et al., 2015).

## 5 Key autophagy regulators in the modulation of OA

Key autophagy regulators commonly involved in the modulation of OA include mTOR, AMPK, sirtuins (SIRT), ULK1, Liver Kinase B1 (LKB1), LC3, and non-coding RNAs (ncRNAs), among others. These factors form an intricate network of interactions, collaboratively regulating autophagy to maintain cartilage homeostasis.

### 5.1 The mTOR-centric autophagy regulator network

mTOR exists in two distinct complexes, mTORC1 and mTORC2, and serves as a negative regulator of autophagy. mTORC1, in particular, is the main regulatory target for autophagy. Upon activation, mTORC1 inhibits the phosphorylation of ULK1 and ATG13 proteins, as well as the activity of transcription factor EB, thereby suppressing autophagy and affecting cartilage metabolism and regulation. The TSC1/TSC2 complex acts as a negative regulator of mTORC1 by inhibiting the activity of Ras-related GTP-binding protein (RAG GTPase), which would otherwise promote mTORC1 activation (Zha et al., 2011). Additionally, stress conditions such as hypoxia induce the production of DNA damage response regulator 1 (REDD1), which further activates the tuberous sclerosis complex (TSC), thereby inhibiting mTORC1 and inducing autophagy (Dennis et al., 2014). Research has shown that REDD1 is highly expressed in normal human cartilage, but its levels are relatively reduced in OA patients, suggesting that the lack of REDD1 in OA exacerbates the severity of the condition (Alvarez-Garcia et al., 2016). Moreover, p53 can upregulate TSC2 expression, enhancing the inhibitory effect of the TSC1/TSC2 complex on mTORC1 (He et al., 2020). Eukaryotic initiation factor 4E-binding protein 1 (4E-BP1) and S6 kinase 1 (S6K1) are downstream effector proteins of mTORC1. mTORC1 phosphorylates 4E-BP1 and S6K1; phosphorylated 4E-BP1 loses its inhibitory effect on protein synthesis, while S6K1 promotes the synthesis of proteins, lipids, and nucleotides, leading to autophagy suppression (Dodd et al., 2015). Additionally, several signaling pathways, including PI3K/AKT, RAS/MAPK, and Hippo, modulate autophagy through mTOR. The PI3K/AKT pathway activates mTORC1 by inhibiting the TSC1/TSC2 complex (Li et al., 2022), while the RAS/MAPK pathway activates MEK and extracellular signal-regulated kinase (ERK), which phosphorylate raptor, a regulatory subunit of mTOR, thus activating mTORC1 and significantly promoting autophagy in OA chondrocytes (Yang et al., 2018). On the other hand, the Hippo pathway, when activated, inhibits yes-associated protein (YAP), thereby suppressing mTORC1 activation and promoting autophagy in OA chondrocytes (Sun et al., 2023).

### 5.2 The AMPK-centric autophagy regulator network

In contrast to mTOR, AMPK serves as a positive regulator of autophagy. The primary mechanisms through which AMPK promotes autophagy are threefold: first, phosphorylated AMPK activates the TSC1/TSC2 complex, thereby inhibiting mTORC1 activity and indirectly promoting autophagy. Second, AMPK directly phosphorylates and activates the ULK1 complex, initiating the formation of autophagosomes. Third, AMPK phosphorylates the transcription factors TFEB and FOXO3; phosphorylated TFEB translocates to the nucleus and binds to the promoters of autophagy and lysosome-related genes, upregulating their expression, while phosphorylated FOXO3 enhances the expression of ATG genes (Lu et al., 2024). AMPK activity is tightly regulated, primarily through phosphorylation. When cellular energy is depleted, ATP consumption increases, leading to elevated AMP/ADP levels and reduced ATP levels. AMP and ADP bind directly to the γ subunit of AMPK, and LKB1 phosphorylates AMPK at the Thr172 site, activating it and inducing autophagy (Wani et al., 2021). In chondrocytes, certain stress signals can lead to elevated intracellular Ca^2+^ levels. Calcium binds with calmodulin, activating CaMKKβ, which in turn phosphorylates AMPK at the Thr172 site, further promoting autophagy (Xiao et al., 2022). Elevated ROS levels can lead to DNA damage, activating ATM kinase, which phosphorylates and activates LKB1, selectively triggering mitochondrial autophagy (Pu et al., 2023). In hypoxic conditions, increased expression of hypoxia inducible factor-1α (HIF-1α) promotes autophagy in chondrocytes, reducing ROS and inflammatory factors, and enhancing cartilage repair capacity. Knockdown of HIF-1α impairs autophagy, exacerbates mitochondrial oxidative damage, and ultimately leads to chondrocyte apoptosis (Lu et al., 2021). SIRT1 and FOXO3a, as downstream signals of AMPK, have been shown to regulate the expression of ATG proteins and control autophagosome formation (Lee et al., 2022). Additionally, SIRT1 promotes the activation of LKB1 and its translocation to the cytoplasm, indirectly facilitating LKB1-mediated phosphorylation and activation of AMPK(Lee et al., 2022). Further studies have revealed that upregulation of SIRT3 expression enhances Parkin-mediated mitochondrial autophagy, alleviating chondrocyte apoptosis and ECM degradation, and improving chondrocyte function (Shi P.-B. et al., 2025). Overexpression of SIRT3 in joints reduces the severity of OA-induced joint damage, suggesting that SIRT3 is an important therapeutic target for OA (Deng et al., 2025). Moreover, SIRT3 protects chondrocytes by maintaining normal mitochondrial function. FOXO3 may also play a pivotal role in cartilage development and maturation, protecting cartilage from OA-induced damage (Wu et al., 2025). AMPK activity is also negatively regulated by phosphatases, the most notable being protein phosphatase 2A and protein phosphatase 2C. Under inflammatory conditions, the activity of these phosphatases may be upregulated, leading to sustained low AMPK activity and inhibited autophagy, thereby exacerbating chondrocyte apoptosis and cartilage degeneration (Magnaudeix et al., 2013).

### 5.3 ncRNA

ncRNAs do not encode proteins, yet they play crucial regulatory roles in cellular processes. Research has shown that ncRNAs are involved in the progression of various diseases, including OA (Zheng et al., 2021), rheumatoid arthritis (Zhu et al., 2024), gouty arthritis (Li X. et al., 2020), and cancer (Chen B. et al., 2022). NcRNAs are typically categorized by their length into lncRNAs and short non-coding RNAs, with the major types being microRNAs (miRNAs), lncRNAs, and circular RNAs (circRNAs). These ncRNAs are increasingly recognized for their regulatory roles in autophagy, a critical process for maintaining the homeostasis of OA cartilage (Zhu et al., 2024). Moreover, accumulating studies have demonstrated that miRNAs, lncRNAs, and circRNAs participate in regulating autophagy in OA.

#### 5.3.1 MiRNAs

MiRNAs are single-stranded non-coding RNA molecules consisting of 19–24 nucleotides that mediate post-transcriptional gene silencing by targeting mRNA, playing pivotal roles in various physiological processes (Diener et al., 2022). Studies have shown that many miRNAs participate in the regulation of autophagy in OA chondrocytes, thereby influencing cartilage degeneration (Table 1). For instance, miR-302d-3p and miR-155 target ULK1, inhibiting the initiation of autophagy in chondrocytes and promoting chondrocyte apoptosis, thus exacerbating cartilage degeneration (Wang et al., 2019). Additionally, several miRNAs influence the progression of cartilage degeneration by regulating ATG proteins. For example, miR-155 targets ATG14, ATG5, and ATG3, leading to defects in chondrocyte autophagy (D’Adamo et al., 2016). miR-128 inhibits the expression of ATG12, suppressing autophagy in chondrocytes and accelerating cartilage degeneration (Lian et al., 2018). miR-375 targets ATG2B, inhibiting chondrocyte autophagy, promoting ERS and chondrocyte apoptosis, and thereby aggravating OA (Li et al., 2020). miR-378 reduces the expression of ATG2A and SOX6, inhibiting autophagy in chondrocytes, thus suppressing chondrogenesis and worsening OA (Shi J. et al., 2025). In summary, miRNAs directly regulate autophagy-related proteins or signaling pathways involved in processes such as autophagy initiation and autophagosome formation, contributing to the regulation of autophagy in OA chondrocytes.

**TABLE 1:** MiRNAs regulating chondrocyte autophagy in OA.

MiRNA	MiRNA expression	Model/Cell types	Effects on autophagy	Target/Signaling pathway	Results	References
miR-302d-3p	Upregulated	In clinical, human OA cartilage In vitro, miR-302d-3p mimics transfected chondrocytes	Inhibit	ULK1	Inhibited proliferation, promoted apoptosis	Wang et al. (2019)
miR-155	Upregulated	In vitro, rapamycin and 2-deoxyglucose induced human chondrocytes	Inhibit	ULK1, FOXO3, ATG14, ATG5, ATG3	Defective autophagy	D’Adamo et al. (2016)
miR-128	Upregulated	In vivo, ACLT surgery rat In vitro, IL-1β-induced rat chondrocytes	Inhibit	ATG12	Accelerated cartilage degeneration	Lian et al. (2018)
miR-375	Upregulated	In vivo, DMM-induced C57BL/6J mice In vitro, mice chondrocytes	Inhibit	ATG2B	Enhanced ERS and apoptosis	Li et al. (2020)
miR-378	Upregulated	In vivo, miR-378 TG mice In vitro, miR-378 TG mouse chondrocytes	Inhibit	ATG2A, SOX6	Inhibited chondrogenesis	Shi et al. (2025a)
miR-20	Upregulated	In vitro, IL-1β-induced rat chondrocytes	Inhibit	ATG10, PI3K/AKT/mTOR	Inhibited chondrocyte activity	He and Cheng (2018)
miR-103-3p	Upregulated	In vitro, IL-1β-induced mice chondrocytes	Promote	PI3K/AKT/mTOR	Reduced apoptosis and ECM degradation	Li et al. (2025a)
miR-7	Downregulated	In vitro, IL-1β-induced C28/I2 chondrocytes In vivo, ACLT surgery rat	Inhibit	PI3K/AKT/mTOR	Exacerbated cartilage degeneration	Zhou et al. (2020)
miR-31-5p	Upregulated	In vitro, IL-1β-induced rat chondrocytes	Promote	SOX4/mTORC1	Enhanced chondrocyte vitality	Xu et al. (2022)
miR-34a-5p	Upregulated	In clinical, cartilage of OA patients	Inhibit	AMPK/mTOR	Inhibited proliferation and migration	Wang et al. (2024a)
miR-411	Upregulated	In vitro, IL-1β-induced C28/I2 chondrocytes	Promote	HIF-1α	Enhanced chondrocyte vitality	Yang et al. (2020)
miR-142-3p	Downregulated	In vitro, mice chondrocytes	Promote	HMGB1	Inhibited cartilage degeneration	Wang et al. (2021)
miR-146b-5p	Upregulated	In vitro, rat chondrocytes	Promote	TRAF6	Protected ECM and chondrocytes	Lou et al. (2023)
miR-199a-5p	Downregulated	In vivo, ACLT surgery rat In vitro, rat chondrocytes	Promote	MAPK4	Protected cartilage and improved OA	Lu et al. (2022)
miR-140-5p	Upregulated	In vitro, human chondrocytes	Promote	FUT1	Protected chondrocytes	Wang et al. (2018)
miR-149	Upregulated	In vitro, human chondrocytes	Promote	FUT1	Protected chondrocytes	Wang et al. (2018)

Furthermore, miRNAs can also modulate the autophagy regulatory network centered on mTOR and AMPK, influencing the process of cartilage degeneration. Studies have shown that miR-20 targets ATG10 while simultaneously promoting the PI3K/AKT/mTOR signaling pathway, thereby inhibiting autophagy in OA chondrocytes (He and Cheng, 2018). Similarly, miR-103-3p and miR-7 also affect the PI3K/AKT/mTOR signaling pathway (Li J. et al., 2025). In IL-1β-induced mouse chondrocytes, the upregulation of miR-103-3p suppresses the PI3K/AKT/mTOR signaling pathway, promoting chondrocyte autophagy and inhibiting ECM degradation. In contrast, both *in vitro* and *in vivo* studies have shown that miR-7 expression is reduced in OA rats, leading to the activation of the PI3K/AKT/mTOR signaling pathway, inhibiting chondrocyte autophagy, and accelerating cartilage degeneration (Zhou et al., 2020). Xu found that miR-31-5p promotes IL-1β-induced chondrocyte autophagy and proliferation in rats by inhibiting the SOX4/mTORC1 axis (Xu et al., 2022). AMPK, a positive regulator of autophagy, and mTOR, a negative regulator, also play a critical role in autophagy regulation. In OA cartilage samples, miR-34a-5p is significantly upregulated while sestrin-2 (SESN2) is notably decreased; this correlates with downregulated AMPK levels and upregulated mTORC1 levels. This suggests that miR-34a-5p inhibits SESN2, suppressing the AMPK pathway and activating the mTOR signaling pathway, thereby inhibiting chondrocyte autophagy (Wang J. et al., 2024). HIF-1α, an upstream protein of AMPK, is upregulated by miR-411, promoting autophagy in chondrocytes (D’Adamo et al., 2016). FOXO3, a downstream protein of AMPK, is suppressed by the upregulation of miR-155, inhibiting autophagy in chondrocytes (Yang et al., 2020). Additionally, *in vitro* cell experiments have demonstrated that miR-142-3p targets high mobility group protein (HMGB1), while miR-146b-5p targets tumor necrosis factor receptor-associated factor 6 (TRAF6), both promoting chondrocyte autophagy and proliferation (Wang et al., 2021; Lou et al., 2023). Upregulation of miR-140-5p and miR-149 targets fucosyltransferase 1 (FUT1), stimulating chondrocyte autophagy (Wang et al., 2018). In conclusion, miRNAs have been clearly shown to participate in the regulation of autophagy in OA chondrocytes, holding potential as new therapeutic targets for OA treatment. However, the specific mechanisms and development of their clinical application still require further investigation.

#### 5.3.2 LncRNA

LncRNAs are RNA molecules longer than 200 nucleotides, characterized by relatively low sequence conservation but high tissue/cell specificity in the expression. Their role is more complex than that of miRNAs, as they can regulate gene expression at various levels-pre-transcriptional, transcriptional, and post-transcriptional - and are involved in a wide range of diseases (Herman et al., 2022). As research into lncRNAs deepens, several have been shown to regulate autophagy in OA chondrocytes, and some have been implicated in the development of OA by interacting with miRNAs to influence the process of cartilage degeneration (Table 2). For instance, in an anterior cruciate ligament transection (ACLT) rat model of OA, the expression of lncRNA-H19 was upregulated. This lncRNA promotes chondrocyte autophagy by targeting miR-148a, a mechanism that helps alleviate cartilage degradation. Conversely, in OA cartilage from human patients, the expression of lncRNA-HOTAIR was also found to be upregulated, but it inhibits autophagy by targeting miR-130a-3p, contributing to cartilage deterioration (Zhou et al., 2025). These examples illustrate how lncRNAs can function through complex interactions with miRNAs to either promote or inhibit autophagy in chondrocytes, highlighting their potential as therapeutic targets in OA. The precise roles of lncRNAs and their mechanisms of action in OA still require further exploration, but their potential is promising for modulating autophagy and influencing cartilage homeostasis (He and Jiang, 2020).

**TABLE 2:** LncRNAs regulating chondrocyte autophagy in OA.

LncRNA	LncRNA expression	Model/Cell types	Effects on autophagy	Target/Signaling pathway	Results	References
lncRNA-H19	Upregulated	In vivo, ACLT surgery rat	Promote	miR-148a	Inhibited inflammation, mitigated cartilage damage	Zhou et al. (2025)
lncRNA-HOTAIR	Upregulated	In clinical, cartilage in OA patients	Inhibit	miR-130a-3p	Promoted apoptosis	He and Jiang (2020)
lncRNA-NEAT1	Downregulated	In vivo, ACLT surgery mice	Promote	miR-122-5p/Sesn2/Nrf2	Promoted proliferation, inhibited apoptosis	Zhang and Jin (2022)
lncRNA-GAS5	Upregulated	In vivo, ACLT surgery rat	Inhibit	miR-144/mTOR	Promoted apoptosis, accelerated OA	Ji et al. (2021)
lncRNA-POU3F3	Downregulated	In vivo, DMM-induced mice In vitro, IL-1β-induced mice chondrocytes	Promote	POU3F3/miR-29a-3p/FOXO3	Inhibited apoptosis and inflammation	Shi et al. (2022)
lncRNA-PVT1	Upregulated	In vitro, IL-1β-induced C28/I2 chondrocytes	Inhibit	miR-27b-3p/TRAF3	Promoted apoptosis and inflammation	Lu et al. (2020)
lncRNA-SNHG1	Upregulated	In vitro, IL-1β-induced chondrocytes In vivo, ACLT surgery mice	Inhibit	PI3K/AKT	Inhibited apoptosis	Wang et al. (2024b)
lncRNA-GCH1	Upregulated	In vitro, human chondrocytes	Promote	PTEN 1/Parkin	Improved mitochondrial function, inhibited cartilage degradation	Zeng et al. (2025)

Furthermore, numerous lncRNAs influence the signaling pathways involving miRNAs that regulate autophagy in chondrocytes. In a mouse model of OA induced by ACLT, lncRNA-NEAT1 promotes chondrocyte autophagy and proliferation, inhibits apoptosis, and alleviates OA symptoms by modulating the miR-122-5p/Sesn2/Nrf2 signaling pathway (Zhang and Jin, 2022). Another study demonstrated that in a rat OA model induced by ACLT, lncRNA-GAS5 inhibits chondrocyte apoptosis, promotes chondrocyte autophagy, and accelerates cartilage degeneration through the miR-144/mTOR axis (Ji et al., 2021). In both *in vitro* and *in vivo* studies, lncRNA-POU3F3 promotes chondrocyte autophagy, suppresses apoptosis, and reduces inflammation by regulating the POU3F3/miR-29a-3p/FOXO3 axis (Shi et al., 2022). Moreover, lncRNA-PVT1 inhibits chondrocyte autophagy in an *in vitro* cellular model via the miR-27b-3p/TRAF3 axis (Lu et al., 2020). Several studies have also revealed that lncRNA-SNHG1 activates the PI3K/AKT signaling pathway both *in vivo* and *in vitro*, suppressing chondrocyte autophagy and accelerating cartilage degeneration (Wang Q. et al., 2024). Elevated levels of lncRNA-GCH1 were found in the cartilage of OA patients, where it activates the PTEN 1/Parkin signaling pathway, promotes mitochondrial autophagy, improves mitochondrial function, and inhibits cartilage degradation (Zeng et al., 2025). Consequently, the regulation of these genes may hold significant potential for gene therapy in OA.

#### 5.3.3 CircRNA

CircRNAs are a class of non-coding RNA molecules that form a closed circular structure through back-splicing of pre-mRNA. Due to the lack of a 5′ cap structure and a 3′ poly(A) tail, circRNAs are resistant to degradation by exonucleases, leading to a much longer half-life compared to linear mRNA, making them highly stable within the cell (Xu et al., 2024). Furthermore, many circRNAs exhibit tissue specificity and disease-relatedness, making them a prominent focus of research in the ncRNA field in recent years. CircRNAs can function as competing endogenous RNAs (ceRNAs), competing with miRNAs to influence the stability or translation of target RNAs, thereby regulating gene expression at the transcriptional level. For example, in the cartilage tissue of OA patients and LPS-induced OA chondrocytes, the expression of circ-MSR is upregulated and interacts with miR-761 to inhibit autophagy in chondrocytes (Xu et al., 2024). Similarly, circ-Pan3 modulates the miR-667-5p/ghrelin axis, inhibiting autophagy in chondrocytes and accelerating cartilage degeneration (Zeng et al., 2021). Additionally, both *in vivo* and *in vitro* studies have confirmed that upregulated ciRS-7 activates the PI3K/AKT/mTOR signaling pathway, inhibiting autophagy in chondrocytes and exacerbating cartilage degeneration (Zhou et al., 2020). Moreover, circRNAs regulate autophagy through their interactions with autophagy-related proteins. Circ-SPG21 interacts with Parkin to promote mitochondrial autophagy (Zhang et al., 2025c), while circ-TBCK affects LC3 to inhibit autophagy, thereby accelerating cartilage degeneration. Other circRNAs involved in the regulation of autophagy in OA chondrocytes are listed in Table 3.

**TABLE 3:** CircRNAs regulating chondrocyte autophagy in OA.

CircRNA	CircRNA expression	Model/Cell types	Effects on autophagy	Target/Signaling pathway	Results	References
circ-0005567	Upregulated	In vitro, IL-1β-induced rat chondrocytes	Promote	miR-495/ATG14	Inhibited apoptosis	Zhang et al. (2020)
circ-MSR	Upregulated	In vitro, OA human cartilage and LPS-induced OA chondrocyte	Inhibit	FBXO21, miR-761	Inhibited chondrocyte activity	Liang et al. (2024)
circ-MELK	Upregulated	In vitro, TBHP-induced human chondrocytes	Inhibit	miR-497-5p, MYD88/NF-κB	Promoted apoptosis	Zhang et al. (2022b)
circ-RHOT1	Upregulated	In clinical, OA human cartilage	Inhibit	miR-142-5p/CCND1	Inhibited activity, promoted apoptosis	Man et al. (2022)
circ-Pan3	Downregulated	In vitro, IL-1β-induced rat chondrocytes	Inhibit	miR-667-5p/ghrelin	Aggravated OA damage	Zeng et al. (2021)
ciRS-7	Upregulated	In vitro, IL-1β-induced C28/I2 chondrocytes In vivo, ACLT surgery rat	Inhibit	PI3K/AKT/mTOR	Exacerbated cartilage degeneration	Zhou et al. (2020)
circ-FOXO3	Upregulated	In vivo, ACLT surgery mice	Promote	FOXO3, PI3K/AKT	Alleviated OA symptoms	Zhao et al. (2022)
circ-SPG21	Upregulated	In vitro, TBHP-induced rat chondrocytes	Promote	SIRT1, P53, Parkin	Promoted mitophagy, alleviated aging	Y. Zhang et al. (2025)
circ-0037658	Upregulated	In vitro, IL-1β-induced CHON-001 cell	Inhibit	LC3, ATG5, BECN1, p62, collagen III, MMP13	Inhibited OA progression	Sui et al. (2021)
circ-TBCK	Downregulated	In vivo, ACLT surgery mice In vitro, IL-1β-induced ATDC5 cell	Inhibit	LC3, SOX9	Accelerated cartilage degeneration	Wang et al. (2025)

## 6 Autophagy-targeted therapeutic strategies for OA

### 6.1 Autophagy agonists

Activating the normal function of autophagy through pharmacological agents is a crucial strategy for treating OA cartilage degeneration. Drugs stimulate autophagy via various pathways, thereby reducing chondrocyte apoptosis, improving mitochondrial function, and decreasing ECM degradation, which helps protect articular cartilage (Table 4). Rapamycin, a selective mTOR inhibitor, directly targets and inhibits the key autophagy regulator mTOR, thus relieving autophagy suppression and improving OA (Bao et al., 2020). Metformin, through its effect on the AMPKα2/SIRT1 axis, activates autophagy and suppresses apoptosis to alleviate cartilage degeneration in mice (Wang et al., 2020). Peroxisome proliferator-activated receptors (PPARs), a family of nuclear receptor proteins involved in inflammation, lipid metabolism, cell division, proliferation, and the regulation of energy homeostasis, play a pivotal role in OA. The PPARδ agonist GW501516 activates PPARδ, inhibits the AKT/mTOR signaling pathway, and induces autophagy, reducing chondrocyte apoptosis and ECM degradation (Sun et al., 2024). Additionally, the PPARγ agonist pioglitazone restores Pink1/Parkin-dependent mitochondrial autophagy, enhances mitochondrial function, inhibits ferroptosis in chondrocytes, and delays the progression of OA (Xue et al., 2023).

**TABLE 4:** Autophagy activators targeting OA.

Type	Drug name	Model/Cell types	Route of treatment	Dosage	Duration	Signal pathways/Mechanisms	References
Autophagy agonist	Rapamycin	In vitro, IL-1β-induced rat chondrocytes	In vitro, cell seeding	In vitro, 100 nM	In vitro, 24 h	mTOR	Bao et al. (2020)
	Metformin	In vitro, IL-1β-induced mice chondrocytes In vivo, DMM surgery mice	In vitro, cell seeding In vivo, intra-articular injection	In vitro,100 nM In vivo,1.65 g/mL	In vitro, 24 h In vivo, 8 weeks	AMPKα/SIRT1	Wang et al. (2020)
	PPARδ agonist	In vitro, IL-1β-induced rat chondrocytes In vivo, DMM surgery rat	In vitro, cell seeding In vivo, intra-articular injection	In vitro, 100 nM In vivo, 100 nM	In vitro, 24 h In vivo, 8 weeks	AKT/mTOR	Sun et al. (2024)
	PPARγ agonist	In vitro, RSL3-induced rat chondrocytes In vivo, rat OA model	In vitro, cell seeding In vivo, oral administration	In vitro, 40 μM In vivo, 20 mg/kg	In vitro, 24 h In vivo, 4 weeks	Pink1/Parkin	Xue et al. (2023)

### 6.2 Natural products modulating autophagy in OA

Research has shown that numerous natural products can regulate the progression of OA by inducing autophagy in chondrocytes (Table 5). Among these, compounds such as baicalin (Yang et al., 2025), angelica sinensis polysaccharide (Xu et al., 2021), resveratrol (Zou et al., 2024), curcumin (Li et al., 2017), mulberroside A (Lu et al., 2023), astragaloside IV (Liu et al., 2017), icariin (Mi et al., 2018), columbianetin (Chen et al., 2022b), mangiferin, chlorogenic acid (Zada et al., 2021), delphinidin (Lee et al., 2020), punicalagin (Kong et al., 2020), sinensetin (Zhou et al., 2021), rhoifolin (Yan et al., 2021), quercetin (Lv et al., 2022), and shikonin (Wang et al., 2022) all enhance autophagy activity by increasing the ratio of autophagy markers LC3-II/LC3-I and the expression of Beclin-1, while inhibiting the expression of the autophagy substrate p62/SQSTM1. These compounds upregulate autophagic activity, thereby protecting chondrocytes from damage.

**TABLE 5:** Natural compounds targeting chondrocyte autophagy.

Drug name	Sources	Model/Cell types	Route of treatment	Dosage	Duration	Signal pathways/Mechanisms	References
Baicalin	*Scutellaria baicalensis*	*In vivo*, ACLT surgery mice	*In vivo*, intra-articular injection	20 mg/kg	6 weeks	NF-κB/PI3K/PINK1	Yang et al. (2025)
		*In vitro*, IL-β-induced human chondrocytes	*In vitro*, cell seeding	20 μM	12 h	miR-766-3p/AIFM1	Li et al. (2020c)
*Angelica sinensis* polysaccharide	*Angelica sinensis*	*In vitro*, SNP-induced human chondrocytes	*In vitro*, cell seeding	50, 200 μg/mL	24 h	ERK1/2	Xu et al. (2021)
Resveratrol	multiple plants	*In vitro*, SW1353 cells *In vivo*, C57BL/6J OA mice	*In vitro*, cell seeding *In vivo*, oral administration	*In vitro*, 50, 500 μM *In vivo*, 22.5, 45 mg/kg/d	*In vitro*, 24 h *In vivo*, 8 or 12 weeks	TLR4/PI3K/AKT	Xu et al. (2019)
		*In vivo*, rat chondrocytes	*In vitro*, cell seeding	50 μM	24 h	SIRT1	Liang et al. (2022)
Curcumin	Curcuma longa	*In vitro*, IL-1β-induced rat chondrocytes *In vivo*, MIA induced rat	*In vitro*, cell seeding *In vivo*, intra-articular injection	*In vitro*,10 μM *In vivo*, 0.5%	*In vitro*, 24 h *In vivo*, 4 weeks	AMPK/PINK1/Parkin	Jin et al. (2022)
		*In vivo*, HFD-induced obesity-related OA in rats	*In vivo*, intra-articular injection	200, 400 μg/kg	4 weeks	miR-34a/Akt/mTOR	Yao et al. (2021)
		*In vitro*, IL-1β-induced rat chondrocytes	*In vitro*, cell seeding	10 μM	24 h	MAPK/ERK1/2	Li et al. (2017)
Mulberroside A	Mori Cortex	*In vitro*, IL-1β-induced mice chondrocytes	*In vitro*, cell seeding	40 μM	24 h	SIRT1	Lu et al. (2023)
Astragaloside IV	Astragalus membranaceus	*In vitro*, IL-1β-induced human chondrocytes	*In vitro*, cell seeding	50 μg/mL	24 h	LC3-II/I, P62/SQSTM1	Liu et al. (2017)
Icariin	*Epimedium*	*In vivo*, ACLT surgery rat	*In vivo*, intra-articular injection	20, 40, 80 mg/kg	4 weeks	PI3K/AKT/mTOR	Tang et al. (2021)
		*In vitro*, TNF-α-induced rat chondrocytes	*In vitro*, cell seeding	10 μM	24 h	NF-κB	Mi et al. (2018)
Columbianetin	*radix angelicae pubescentis*	*In vitro*, LPS-induced mice chondrocytes	*In vitro*, cell seeding	40 μg/mL	24 h	SGK1	Chen et al. (2022b)
Mangiferin	*Mangifera indica* L	*In vivo*, DMM surgery mice	*In vivo*, oral administration	50 mg/kg	8 weeks	AMPK	Li et al. (2019)
Chlorogenic acid	polyphenolic compound	*In vitro*, H_2_O_2_-induced human C28/I2 chondrocytes	*In vitro*, cell seeding	250 μM	24 h	NRF2/NF-κB	Zada et al. (2021)
Delphinidin	multiple plants	*In vitro*, H_2_O_2_-induced human C28/I2 chondrocytes	*In vitro*, cell seeding	40 μM	24 h	NRF2/NF-κB	Lee et al. (2020)
Punicalagin	*Punica granatum*	*In vitro*, TBHP-induced mice chondrocytes *In vivo*, DMM surgery mice	*In vitro*, cell seeding *In vivo*, oral administration	*In vitro*, 50 μg/mL *In vivo*, 20 mg/kg	*In vitro*, 24 h *In vivo*, 8 weeks	p62/LC3 II/I	(Kong et al., 2020)
Sinensetin	Citrus fruits	*In vitro*, TBHP-induced mice chondrocytes *In vivo*, DMM surgery mice	*In vitro*, cell seeding *In vivo*, oral administration	*In vitro*, 40 μM *In vivo*, 50 mg/kg	*In vitro*, 24 h *In vivo*, 8 weeks	AMPK/mTOR/LC3	Zhou et al. (2021)
(−)-Epigallocatechin 3-gallate	green tea	*In vivo*, ACLT surgery rat	*In vivo*, intra-articular injection	10 μM	5 weeks	mTOR/beclin-1/LC3/p62	Huang et al. (2020)
Rhoifolin	Rhus succedanea	*In vitro*, IL-1β-induced rat chondrocytes *In vivo*, ACLT surgery rat	*In vitro*, cell seeding *In vivo*, intra-articular injection	*In vitro*, 20 μM *In vivo*, 20 μM	*In vitro*, 24 h *In vivo*, 8 weeks	P38/JNK, PI3K/AKT/mTOR	Yan et al. (2021)
Quercetin	multiple plants	*In vitro*, IL-1β-induced rat chondrocytes	*In vitro*, cell seeding	8 μM	24 h	TSC2-RHEB-mTOR	Lv et al. (2022)
Shikonin	*Lithospermum*	*In vitro*, IL-1β-induced human chondrocytes	*In vitro*, cell seeding	5, 10, 20 μmol/L	24 h	Beclin-1/LC3II/LC3I	Wang et al. (2022)

Moreover, Icariin, rhoifolin, and mulberroside A regulate chondrocyte autophagy by mediating autophagy-related signaling pathways such as PI3K/AKT/mTOR (Lu et al., 2023; Tang et al., 2021; Yan et al., 2021). Curcumin, mangiferin, and sinensetin enhance autophagy through the activation of the AMPK signaling pathway (Jin et al., 2022; Li et al., 2019; Zhou et al., 2021). Chlorogenic acid and delphinidin promote the initiation of autophagy and suppress cartilage degeneration by activating pathways involving Nrf2 and NF-κB (Zada et al., 2021).

In addition, Baicalin activates autophagy via the miR-766-3p/AIFM1 axis, thereby protecting human OA chondrocytes (Li Z. et al., 2020). Curcumin upregulates autophagy-related pathways such as AKT/mTOR by inhibiting miR-34a, thus safeguarding chondrocytes (Yao et al., 2021). Columbianetin improves OA by activating chondrocyte autophagy through the inhibition of serum and glucocorticoid-induced protein kinase 1 (SGK1) expression (Chen et al., 2022b). Punicalagin enhances the phosphorylation of ULK1, promoting an autophagic state and improving the dysfunctional autophagic flux in chondrocytes (Kong et al., 2020).

### 6.3 Emerging strategies for targeting autophagy in the treatment of cartilage degeneration

#### 6.3.1 Exosome-mediated autophagy regulation

Exosomes derived from mesenchymal stem cells serve as natural nanocarriers, characterized by excellent biocompatibility, low immunogenicity, and inherent cartilage-targeting properties, making them an ideal platform for delivering autophagy modulators in OA therapy. On one hand, exosomes are rich in miRNAs that can regulate autophagy; for instance, miR-98-5p carried by exosomes activates protective autophagy by targeting transcription factor 6 (Xu and Zhang, 2025). On the other hand, exosomes function as delivery systems, capable of loading small molecule autophagy drugs or mRNA via techniques such as electroporation, ultrasound, or co-incubation, thereby enabling targeted drug delivery. Moreover, surface modification of exosomes with cartilage-targeting peptides allows for the active, targeted delivery of drugs to the affected cartilage, significantly enhancing local drug concentration while minimizing systemic exposure (Kuang et al., 2025).

#### 6.3.2 Biomaterial scaffolds synergistically activating autophagy to construct a cartilage repair microenvironment

Intelligent hydrogels or nanofiber scaffolds can serve as carriers for autophagy modulators. By encapsulating small molecule autophagy activators within responsive biomaterials, drugs can be released on demand at the site of pathology. When inflammation or an acidic microenvironment arises within the OA joint cavity, the material degrades more rapidly, releasing the drug to activate autophagy, thereby suppressing inflammation and protecting chondrocytes (He et al., 2025). The properties of the material itself can also indirectly influence cellular autophagy. Studies have shown that biomaterials with specific topological structures or mechanical properties can affect autophagy levels through mechanotransduction signals (Feng et al., 2025). By utilizing such materials, a synergistic effect between the materials and the loaded drugs can be achieved, promoting chondrocyte anabolic metabolism and maintaining homeostasis more effectively.

#### 6.3.3 Artificial intelligence and machine learning in predicting autophagy-drug interactions

The traditional drug development model, when applied to the complex and dynamic process of autophagy, significantly increases both the cost and time required for research. Artificial intelligence (AI) and machine learning (ML), by mining vast biomedical datasets, have the potential to accelerate the development of autophagy modulators. Initially, deep learning algorithms can quickly identify lead compounds with potential regulatory activity by analyzing the chemical structures and bioactivity data of known autophagy modulators. Furthermore, AI can design novel molecules with entirely new structures based on specific targets, thereby enhancing the efficiency of drug discovery. Additionally, considering the heterogeneity of OA patients, AI algorithms can identify distinct OA subtypes, aiding in the selection of the most suitable patient populations for clinical trials, thus advancing personalized medicine (Zitnik et al., 2018; Stokes et al., 2020). Therefore, the future of OA research requires the establishment of a more comprehensive multi-omics database for autophagy and the development of more interpretable AI models to better understand the biological logic behind their predictions.

## 7 Discussion and future perspectives

In contrast to earlier reviews that focused predominantly on isolated autophagy mechanisms or therapeutic approaches, this review provides an integrated analysis spanning from molecular pathways to translational strategies. While autophagy, as a core regulator of chondrocyte homeostasis, plays a dual role in cartilage degeneration and offers a critical perspective for novel therapeutic development, our synthesis uniquely bridges several emerging topics—such as the autophagy–circadian rhythm axis, lipid-induced autophagy dysfunction, and ncRNA-mediated regulatory networks—which have not been comprehensively covered in previous OA reviews. We further critically evaluate the stage-specific transition of autophagy from a protective to a detrimental process and discuss innovative intervention platforms including exosome-based delivery, biomaterial scaffolds, and AI-driven drug design. By systematically examining autophagy’s biological processes, its interactions with cartilage degeneration, and targeted intervention strategies, this review not only highlights unresolved issues requiring further mechanistic, technological, and translational investigation but also establishes a more holistic and clinically relevant perspective on targeting autophagy in OA.

From a mechanistic standpoint, the functional transition of autophagy at different stages of OA has not yet been fully elucidated. A core and unresolved question is how to identify the precise molecular switches that regulate the shift of autophagy from a protective mechanism in the early stages of the disease to a detrimental process in advanced stages. Studies have confirmed that early-stage activation of autophagy in OA can protect chondrocytes by clearing damaged mitochondria and alleviating endoplasmic reticulum stress (ERS), whereas late-stage autophagy dysfunction exacerbates cartilage degeneration (Varghese et al., 2022). Emerging evidence suggests that this transition is not merely a linear decline in autophagic flux, but rather a dysregulation of specific nodes within the autophagy-lysosomal network. For example, age-related lysosomal dysfunction leads to decreased autophagosome degradation efficiency, yet the specific molecular mechanisms and causal relationships between lysosomal dysfunction and the decline in autophagy remain unclear (Aman et al., 2021). Furthermore, the complex interaction network between autophagy, cellular senescence, inflammation, and oxidative stress requires deeper analysis. For instance, activation of the NLRP3 inflammasome inhibits protective autophagy but is exacerbated by autophagy defects, creating a vicious cycle whose underlying mechanisms still need to be explored through advanced techniques such as single-cell sequencing and protein interaction analysis (López-Otín et al., 2023).

Several candidate biomarkers and signaling hubs deserve attention in this transition. First, lysosomal dysfunction, marked by decreased activity of cathepsins and reduced expression of LAMP1/LAMP2, represents a critical checkpoint (Lawrence and Zoncu, 2019). When lysosomal efficiency declines due to aging or sustained inflammatory stress, autophagosomes continue to accumulate despite ongoing initiation, leading to the release of undegraded cargo (e.g., calcium crystals, inflammatory mediators) that directly promote cartilage calcification and inflammation (Aman et al., 2021). Second, the mTOR-AMPK signaling axis plays a dynamic regulatory role: transient activation of AMPK or inhibition of mTOR can induce protective autophagy, whereas chronic dysregulation (e.g., sustained mTORC1 activity under metabolic stress) may lead to impaired autophagosome clearance and selective blockade of mitophagy (Zhao and Klionsky, 2011). Third, the integrity of the ULK1 complex and the processing of LC3-II to its lipidated form may serve as functional indicators—early OA often exhibits compensatory upregulation of these markers, whereas late-stage OA is characterized by their decline and the accumulation of p62/SQSTM1, reflecting arrested autophagic flux (Yang and Klionsky, 2021). Additionally, circadian clock genes (e.g., BMAL1) and specific miRNAs (e.g., miR-34a-5p, miR-155) have emerged as upstream regulators that fine-tune the rhythm and efficiency of autophagy; their dysregulation may precede the functional decline of autophagy.

At the clinical translation level, current targeted autophagy therapies for OA face several interconnected challenges that can be structured into three main categories: biological complexity, technical delivery, and clinical evaluation. (1) Biological Complexity Challenges. A primary hurdle lies in the inherent specificity and context-dependency of autophagy regulation. OA is a highly heterogeneous disease, with significant variations in etiology, molecular pathways, and disease stage among patients (Cai et al., 2025). Consequently, the simple strategy of “autophagy activation” may not be universally beneficial and could even be detrimental in advanced stages where autophagic flux is fundamentally impaired. This biological complexity is compounded by the limitations of current preclinical models. Most research relies on animal models or monolayer chondrocyte cultures, which, despite offering controllability, fail to fully recapitulate the human OA microenvironment, including the interplay between different joint tissues and the chronic, low-grade inflammation (Jeon et al., 2018). This gap contributes to the frequent failure of promising preclinical findings in human clinical trials. Developing more physiologically relevant models, such as organ-on-a-chip systems, 3D cartilage explants, or patient-derived induced pluripotent stem cell (iPSC) models, is crucial to better predict therapeutic efficacy and understand stage-specific responses (Tang et al., 2024). (2) Technical Delivery Challenges. Effective delivery of autophagy modulators to their intended site of action within the joint represents a significant technical barrier. The dense, avascular ECM of articular cartilage severely impedes drug penetration, often resulting in subtherapeutic concentrations at the chondrocyte level (Mourya et al., 2023). Moreover, systemic administration raises the risk of off-target effects due to the ubiquitous role of autophagy in other tissues, potentially causing severe side effects. Overcoming this requires the development of sophisticated, cartilage-targeted delivery systems. Strategies such as nanoparticle carriers functionalized with cartilage-homing peptides, exosome-based delivery platforms, or intra-articularly injectable sustained-release hydrogels are under exploration to enhance local bioavailability while minimizing systemic exposure (Zhao et al., 2024). (3) Clinical Evaluation Challenges. The progression towards personalized, stage-stratified therapy is hampered by the lack of reliable, non-invasive biomarkers. Currently, there are no validated clinical tools to accurately assess *in vivo* autophagic activity within the joints of OA patients (Roemer et al., 2022). This deficiency creates major obstacles in clinical trial design: it complicates patient stratification (selecting those most likely to benefit from a specific autophagy-modulating strategy) and hinders the precise pharmacodynamic monitoring of drug effects on the intended target pathway. Future efforts must focus on discovering and validating biomarkers of autophagic flux, potentially through advanced imaging techniques, proteomic/lipidomic analysis of synovial fluid, or the detection of autophagy-related cargo in circulating extracellular vesicles. Addressing these intertwined challenges—through advanced disease modeling, smart delivery technologies, and biomarker development—is essential for translating the promise of autophagy modulation into safe, effective, and personalized therapies for OA patients.

Future research should adopt a phased, translation-focused approach to bridge the gap between preclinical discovery and clinical application. In the preclinical stage, efforts should prioritize the development of advanced disease models—such as organoids and 3D cartilage explants—that more faithfully recapitulate the pathological microenvironment of human OA. These models will facilitate the identification and optimization of autophagy-modulating compounds with higher translational potential. In the clinical translation stage, emphasis should shift toward enabling technologies, including intelligent drug delivery systems (e.g., exosomes functionalized with cartilage-targeting peptides) for precise delivery of autophagy modulators, as well as the discovery and validation of stage-specific biomarkers and non-invasive imaging tools to monitor autophagic activity in patients. Additionally, the integration of multi-omics data to map OA-specific autophagy networks, coupled with AI and machine learning approaches to predict autophagy–drug interactions and accelerate drug discovery, will be crucial for advancing personalized, stage-stratified OA therapies.

In summary, the dual role of autophagy in OA cartilage degeneration provides innovative therapeutic targets for OA treatment. However, its complex mechanisms and the clinical translation bottleneck still require breakthroughs through interdisciplinary approaches. Future research should focus on the precise regulation of autophagy activity and the realization of cartilage-targeted therapies as core objectives. This will help translate fundamental research findings into clinical applications, ultimately offering safe and effective novel treatment options for OA patients.

## Glossary

ECM: extracellular matrix
OA: osteoarthritis
ERS: endoplasmic reticulum stress
CMA: chaperone-mediated autophagy
ATG: autophagy-related genes
PE: phosphatidylethanolamine
LC3: microtubule‐associated protein light‐chain 3 protein
AMPK: AMP-activated protein kinase
ROS: reactive oxygen species
FFAs: free fatty acids
NLRP3: NOD-like receptor pyrin domain-containing 3
Runx2: runt-related transcription factor 2
BMAL1: brain and muscle arnt-like protein 1
SASP: senescence-associated secretory phenotype
SIRT: sirtuins
LKB1: liver kinase B1
ncRNA: non-coding RNA
REDD1: DNA damage response regulator 1
TSC: tuberous sclerosis complex
4E-BP1: 4E-binding protein 1
S6K1: S6 kinase 1
ERK: extracellular signal-regulated kinase
HIF-1α: hypoxia inducible factor-1α
miRNA: microRNA
lncRNA: long non-coding RNA
circRNA: circular RNA
SESN2: sestrin-2
HMGB1: high mobility group protein
TRAF6: tumor necrosis factor receptor-associated factor 6
FUT1: fucosyltransferase 1
ACLT: anterior cruciate ligament transection
PPAR: peroxisome proliferator-activated receptors
SGK1: serum and glucocorticoid-induced protein kinase 1
AI: artificial intelligence
ML: machine learning
